# Psychological resilience as a mediator between caring attitudes and abuse risk in Chinese dementia family caregivers: a cross-sectional study

**DOI:** 10.3389/fpsyg.2026.1869662

**Published:** 2026-07-07

**Authors:** Shihua Pan, Yueling Wu, Fang Chen, Xiaomin Ding, Jing Mei, Huimin Sun

**Affiliations:** 1 Department of General Medicine, Zhongnan Hospital of Wuhan University, Wuhan, China; 2 Department of Scientific Research, Zhongnan Hospital of Wuhan University, Wuhan, China; 3 Department of Neurology, Zhongnan Hospital of Wuhan University, Wuhan, China; 4 Department of Geriatrics, Zhongnan Hospital of Wuhan University, Wuhan, China; 5 Department of Neuropsychology, Zhongnan Hospital of Wuhan University, Wuhan, China; 6 Clinical Medical Research Center for Dementia and Cognitive Impairment of Hubei Province, Wuhan, China

**Keywords:** abuse risk, caring attitudes, China, dementia, family caregivers, mediation analysis, psychological resilience

## Abstract

**Introduction:**

Family caregivers of people with dementia face an elevated risk of abusive behaviors. However, the psychological association linking caring attitudes to abuse risk—specifically the indirect role of resilience—remains underexplored in non-Western contexts. This cross-sectional study examined whether psychological resilience shows an indirect association in this relationship among Chinese dementia family caregivers.

**Method:**

This cross-sectional study recruited 133 family caregivers from a dementia research center in central China (June 2023–May 2024). Participants completed measures of resilience, caring attitudes, and abuse risk. Data were analyzed using correlations, binomial logistic regression, and mediation analysis (PROCESS Macro, Model 4; 5,000 bootstrap samples).

**Results:**

66.2% of caregivers screened positive for abuse risk. More positive caring attitudes and higher resilience were each significantly correlated with lower abuse risk (*r* = −0.71 and *r* = −0.43, *p* < 0.001). When abuse risk was defined using a CASE cut-off (≥3 vs. <3), more positive caregiving attitudes were associated with significantly lower abuse risk (OR = 0.11, 95% CI: 0.04–0.27). These results remained consistent across subgroups, with no notable multicollinearity (all VIFs <5). Mediation analysis indicated that resilience had a significant partial indirect effect. Notably, the strength of this indirect effect varied across attitudinal dimensions: it was strongest for maintaining positivity (29.2%) and managing reluctance (22.6%), whereas the association from acute emotional distress (e.g., burnout) to abuse risk remained largely direct.

**Conclusion:**

Psychological resilience shows a significant partial and differential indirect association between caring attitudes and abuse risk. These findings suggest a dual-focus intervention—addressing acute distress while building resilience—as a hypothesis-generating strategy for abuse prevention in China’s filial-piety-based dementia care.

## 1 Introduction

Elder abuse is a critical global public health issue (Gimeno et al., 2021). According to the World Health Organization (WHO), one in six older adults worldwide has experienced abuse, encompassing psychological, financial, and physical abuse as well as neglect (Shen et al., 2025). Older adults living with dementia are at a disproportionately heightened risk due to their advanced age, cognitive decline, functional impairments, and communication barriers (de Oliveira et al., 2025). The prevalence of abuse in this population is alarmingly high, with a recent comprehensive report indicating that 42.6% of people with dementia experience some form of abuse (Shen et al., 2025). Notably, psychological abuse is the most prevalent subtype, accounting for 44.8% of these cases (Shen et al., 2025). This risk is further amplified within the familial caregiving contexts prevalent across many Asian societies, where family members are the primary, and often sole, providers of long-term care (Zhao Y. et al., 2023).

In China, the confluence of rapid population aging and a deeply rooted familial care culture presents a distinct and escalating challenge. The number of people living with dementia in China is projected to reach 28 million by 2050 (Han et al., 2025). Anchored in the Confucian ethic of filial piety (xiao), a core traditional value emphasizing adult children’s duty to care for and respect aging parents, 94.3% of care is provided at home by family members, primarily spouses or adult children (Chen et al., 2025; Yu et al., 2025). While this model upholds cherished cultural values and provides a sense of familial duty, it often places an immense and solitary burden on caregivers. They typically undertake this complex and demanding role with minimal formal training, inadequate systemic support, and under considerable social and economic pressure (Yu et al., 2025; Xiang et al., 2025). This environment fosters severe caregiver strain, including chronic psychological distress and burnout, which are well-established proximal risk factors for the initiation or escalation of elder abuse (Wang et al., 2019; Pan et al., 2023). The consequences of such abuse are profound, causing significant harm to the care recipient’s physical and psychological well-being, potentially accelerating cognitive and functional decline, and severely diminishing the quality of life for both members of the care dyad (Shaw et al., 2025; LeRolland et al., 2025). Elucidating the psychological mechanisms underlying abuse risk is therefore imperative for developing effective prevention strategies.

Within this high-stress context, understanding and bolstering caregivers’ internal psychological resources are paramount for prevention. Caring attitudes—encompassing cognitive appraisals and emotional responses toward the care recipient and the caregiving role—are fundamental to care quality and behavioral outcomes (Bannon et al., 2021). Our prior work has refined this understanding, showing that distinct aspects of empathy (e.g., cognitive versus personal distress) relate differentially to caregiver mental health (Mei et al., 2025), highlighting that specific emotional components of attitudes are critical and may have divergent pathways to outcomes. Psychological resilience, defined as the capacity to adapt positively to adversity, serves as another crucial protective factor (Wang et al., 2025). In collectivist cultures like China, resilience may be uniquely expressed and cultivated through concepts of familial duty and quiet endurance (rennai) (Yu et al., 2025; Yang et al., 2025), which may buffer against caregiving stress and mitigate abuse risk. However, the mechanism through which these two key psychological resources—caring attitudes and resilience—interact to influence abuse risk remains unexplored.

Although caring attitudes and psychological resilience have been independently linked to abuse risk, their interrelated pathways—particularly the potential mediating role of resilience—remain underexplored within Asia’s dominant family-based care paradigm (Lee et al., 2024). The Stress Process Model suggests that resilience may act as a bridge, translating caregivers’ appraisals (e.g., attitudes) into behavioral outcomes (Han et al., 2022). The urgency of testing this specific pathway is heightened by practical realities. Our prior work has identified a high-stress caregiver profile strongly linked to difficult-to-change factors like low income and lack of support (Chen et al., 2025). For these vulnerable individuals, directly improving socioeconomic conditions is often not feasible in the short term. Therefore, examining whether resilience mediates the link between attitudes and abuse risk is crucial. This study aims to move beyond simple associations by testing this theory-based mechanism. By clarifying how a changeable internal resource influences behavior, it seeks to identify a practical intervention target, even in resource-limited settings.

Therefore, guided by our prior findings on caregiver empathy (Mei et al., 2025) and high-risk profiles (Chen et al., 2025), this study empirically tests the proposed mediating role of psychological resilience in the relationship between caring attitudes and abuse risk among Chinese family dementia caregivers. Unlike prior research that focused on caregiver burden or coping styles (Pan et al., 2023), the present study examines multi-dimensional caring attitudes as the primary predictor, which provides a more nuanced, clinically actionable perspective. Based on the Stress Process Model and previous empirical evidence, we proposed the following hypotheses: (H1) More positive caring attitudes are associated with lower abuse risk (total effect). (H2) Psychological resilience is positively associated with caring attitudes (*a*-path). (H3) Psychological resilience is negatively associated with abuse risk (*b*-path). (H4) Psychological resilience mediates the relationship between caring attitudes and abuse risk, with the indirect effect (*a* × *b*) being significant. Furthermore, given the multidimensional nature of caring attitudes, we explored whether the strength of this indirect effect varies across attitudinal dimensions (exploratory). By elucidating this mechanism within China’s unique socio-cultural context—where family care is both a cultural ideal and a source of potential strain—this research seeks to inform the development of culturally resonant and psychologically nuanced support strategies. The ultimate goal is to help prevent abuse and enhance the well-being of families undertaking the vital role of dementia care at home.

## 2 Methods

### 2.1 Study design

A cross-sectional correlational study was conducted to identify the mediating effect of psychological resilience on the association of caring attitudes with the risk of abusive behavior among Chinese family caregivers of people with dementia.

### 2.2 Setting and sample

This study employed purposive sampling to recruit family caregivers of patients with dementia. Participants were recruited from the Clinical Medical Research Center for Dementia and Cognitive Impairment of XX Province at XX Hospital of XX University, a leading tertiary hospital in central China. As a major metropolis with a population exceeding 12 million, Wuhan reflects China’s socio-demographic diversity (Wuhan Municipal People’s Government, 2023). Participants were consecutively enrolled from the research center between June 2023 and May 2024, ensuring geographic and clinical diversity within a single-site framework. The inclusion criteria were: (1) being the primary unpaid family caregiver of an individual formally diagnosed with dementia (China Guideline Writing Group for Dementia and Cognitive Impairment, 2018); (2) having provided primary daily care for at least 1 month, and only one primary caregiver per person with dementia (i.e., per household) was enrolled. (3) Being aged 18 years or older; (4) possessing adequate cognitive and communicative abilities to complete the assessment; and (5) providing informed consent to participate. The exclusion criteria included: (1) caregivers with severe physical or mental illnesses; (2) those who had experienced a major stressful life event unrelated to caregiving within the past 6 months (e.g., bereavement, divorce, job loss). According to Kendall’s sample size calculation method (Fang and Lu, 2002), the sample size was considered to be 5–10 times the number of independent variables, and the number of independent variables included in this study was 18 (9 general information questionnaires, 3 dimensions of the Connor-Davidson Resilience Scale, 5 dimensions of the Dementia Carer Attitude Questionnaire, and 1 dimension of the Caregiver Abuse Screen). Considering 20.0% of the questionnaires would be invalid, the sample size to be included was 108–216. During the one-year study period, a total of 277 eligible caregiver–patient dyads were estimated based on hospital records of diagnosed dementia cases and screening for inclusion criteria. The final effective sample size of the present study was acceptable at 133, yielding a response rate of 48.01%. A post-hoc power analysis using G*Power (Fritz and MacKinnon, 2007; Schoemann et al., 2017) with *α* = 0.05, *N* = 133, and an estimated effect size of *f*^2^ = 0.15 yielded observed power values ranging from 0.82 to 0.89, exceeding the recommended benchmark of 0.80, indicating adequate power to detect the hypothesized indirect effects.

### 2.3 Measurement

Permission to use all questionnaires in this study was obtained from the copyright holders via email.

#### 2.3.1 Demographics information

We collected data on the following demographic and caregiving characteristics of participants: type of dementia in the care recipient, severity/stage of dementia in the care recipient, caregiver gender, age, caregiving duration, relationship to the care recipient, educational level, monthly household income, self-rated health status, and availability of caregiving assistance.

#### 2.3.2 Connor-Davidson Resilience Scale

The psychological resilience of caregivers was assessed using the 25-item Connor-Davidson Resilience Scale (CD-RISC), employing the validated Chinese version (Connor and Davidson, 2003; Wu et al., 2017). This scale comprises three dimensions: tenacity, optimism, and strength. Items are rated on a 5-point Likert scale from 0 (never) to 4 (always). Total scores range from 0 to 100, with higher scores indicating greater psychological resilience. Following the interpretation guidelines for the Chinese version, scores can be categorized as low (<60), moderate (60–80), or high (>80) (Wu et al., 2017). In the present sample, the scale demonstrated good internal consistency (Cronbach’s *α* = 0.77).

#### 2.3.3 Dementia Carer Attitude Questionnaire

Caring attitudes were assessed using the validated Chinese version of the 13-item Dementia Carer Attitude Questionnaire (DCAQ) (Liu et al., 2014). This scale comprises five dimensions: positive factors (3 items), burnout factors (4 items), external emotional factors (2 items), negative factors (2 items), and resistance factors (2 items). Items are rated on a 4-point Likert scale from 1 (completely disagree) to 4 (completely agree). For scoring, only the positive factor dimension is reverse-coded (i.e., higher subscale scores indicate more negative attitudes), whereas the other four dimensions are scored positively (i.e., higher subscale scores indicate more positive attitudes). In the total score calculation, the reversed positive factor scores are summed with the original scores of the other four dimensions, so that higher total scores (range: 13–52) indicate more positive overall attitudes toward caregiving (e.g., greater patience, lower burnout, and resistance) (Fang and Lu, 2002). In the current sample, the DCAQ demonstrated acceptable internal consistency (Cronbach’s *α* = 0.79).

#### 2.3.4 Caregiver Abuse Screen

The risk of abusive behavior was assessed using the 8-item Caregiver Abuse Screen (CASE) (Reis and Nahmiash, 1998; Feng and Liu, 2010). This self-report tool screens for potential abusive behaviors, including physical, psychological, and neglect-related items. All items are dichotomous (yes/no), with each “yes” response scored as 1 point, yielding a total score ranging from 0 to 8. Following the established cutoff, a total score of ≥3 indicates a positive screen for abuse risk (Feng and Liu, 2010). The Chinese version of the CASE has demonstrated good validity and reliability (Feng and Liu, 2010). In the current study, the scale showed excellent internal consistency (Cronbach’s *α* = 0.86).

### 2.4 Data collection

Data were collected between June 2023 and May 2024 by trained researchers adhering to a standardized protocol. After potential participants were approached at the research center, the study’s purpose, procedures, and confidentiality safeguards were explained in detail, and written informed consent was obtained. Data collection was conducted primarily through face-to-face interviews using paper-based questionnaires at the center. Specifically, 67 paper questionnaires were distributed. For participants with limited literacy, face-to-face interviews were conducted in which trained interviewers read each item aloud and recorded responses without providing additional explanations that might lead or influence the participants’ responses, following a standardized neutral protocol. For participants who needed to leave before completing the survey, data were collected remotely via the secure online platform Wenjuanxing^1^, administered through telephone or WeChat follow-up to maximize the response rate; 78 online questionnaires were distributed. To ensure data quality, the online platform was configured to require responses to all items and to permit only one submission per IP address or device. Additional quality control measures included checks for patterned responses (e.g., straight-lining). Unfinished face-to-face interviews were excluded as invalid cases. Paper questionnaires were double-entered by two independent researchers into an Excel database, and any discrepancies were resolved by re-checking the original questionnaires. Questionnaire completion required approximately 10–20 min per participant. Each participant received a small gift worth 5 RMB (a pack of tissues) as a token of appreciation upon completion. Of the 145 eligible caregivers invited (78 online, 67 face-to-face), 12 were excluded (8 online due to patterned responses, and 4 face-to-face due to incomplete interviews), 133 were deemed valid (70 online, 63 face-to-face), yielding a 91.7% response rate.

### 2.5 Data analyses

Data were analyzed using SPSS 26.0. Continuous variables with a normal distribution are presented as mean ± standard deviation, while those with a non-normal distribution are reported as median and interquartile range (P_25_, P_75_). Categorical variables are summarized as frequency and percentage (%). Pearson correlation analysis was conducted to examine bivariate relationships. One-way ANOVA was used to compare abuse risk across groups. To test the proposed mediation model, we used the PROCESS macro (Model 4) developed by Hayes, with bias-corrected bootstrap confidence intervals based on 5,000 resamples. All tests were two-tailed. Statistical significance was set at *α* = 0.05 (Preacher and Hayes, 2004). The CASE was treated as a continuous variable in primary analyses to preserve variance and statistical power. As a sensitivity analysis, logistic regression was performed using the dichotomized CASE cutoff (≥3 vs. <3). All mediation models were adjusted for the following dichotomized caregiver demographic covariates: gender (0 = male, 1 = female), age (0 = ≤60 years, 1 = >60 years), education (0 = middle school or below, 1 = high school or above), monthly household income (0 = <5,000 RMB, 1 = ≥5,000 RMB), caregiving duration (0 = ≤1 year, 1 = >1 year), relationship to the care recipient (0 = non-spouse, 1 = spouse), caregiving assistance (0 = no, 1 = yes), and self-rated health (0 = fair/poor, 1 = good). In addition, dementia stage (coded 1, 2, 3 for early, middle, and late) was entered as a covariate in supplementary mediation models as a sensitivity analysis. This study followed the STROBE guidelines for cross-sectional studies (von Elm et al., 2007).

## 3 Results

### 3.1 Characteristics of participants

The sample consisted of 133 family caregivers of people with dementia. Among the care recipients, 73 (54.9%) had a diagnosis of Alzheimer’s disease. In terms of dementia stage, 40 (30.1%) were at the early stage, 62 (46.6%) at the middle stage, and 31 (23.3%) at the late stage. Most caregivers were female (*n* = 88, 66.2%), with a mean age of 60.71 years. The distribution of caregiving duration was: 1–3 months, 15 (11.3%); 3 months to 1 year, 38 (28.6%); 1–3 years, 45 (33.8%); and >3 years, 35 (26.3%). Notably, a total of 80 caregivers (60.1% of the sample) had been providing care for more than 1 year. Over half of the caregivers were spouses of the care recipient (*n* = 77, 57.9%). In terms of education, 72 caregivers (54.2%) had completed high school or above. Slightly more than half (*n* = 74, 55.6%) reported a monthly household income of ≥5,000 RMB, which was higher than the average monthly disposable income per capita of urban residents in Hubei Province during the study period (approximately 3,749 RMB), representing a middle-to-upper income level (Hubei Provincial Bureau of Statistics and National Bureau of Statistics Hubei Survey Team, 2024). Seventy-one caregivers (54.1%) reported no caregiving assistance. Most participants (*n* = 84, 63.2%) rated their overall health as “good.” One-way ANOVA revealed that abuse risk differed significantly across dementia stages (*F* (2, 130) = 4.17, *p* = 0.018, *ɳ*^2^ = 0.06). Detailed sociodemographic characteristics are presented in Table 1.

**Table 1:** Social-demographic data of the family caregivers of people with dementia (*N* = 133).

Variable		Number (*n*)	Composition ratio (%)
Type of dementia in care recipient	Vascular dementia	41	30.8
	Alzheimer’s disease	73	54.9
	Mixed dementia	16	12
Severity/stage of dementia in care recipient	Early-stage dementia	40	30.1
	Middle-stage dementia	62	46.6
	Late-stage dementia	31	23.3
	Other types of dementia	3	2.3
Gender	Male	45	33.8
	Female	88	66.2
Age	≤50	30	22.6
	51–60	48	36.1
	61–80	46	34.6
	≥81	9	6.8
Caregiving duration	1–3 months	15	11.3
	3 months–1 year	38	28.6
	1 year–3 years	45	33.8
	>3 years	35	26.3
Relationship to the care recipient	Spouse	77	57.9
	Children	48	36.1
	Siblings	3	2.3
	Others	5	3.8
Education level	Elementary and below	21	15.8
	Middle school	40	30.1
	High school	36	27.1
	Junior college and above	36	27.1
Monthly household income	≤2000	9	6.8
	2000–5000	50	37.6
	≥5000	74	55.6
Availability of caregiving assistance	Yes	61	45.9
	No	71	54.1
Self-rated health status	General	10	7.5
	Fair	39	29.3
	Good	84	63.2

### 3.2 Levels of psychological resilience, caring attitudes, and abuse risk

For DCAQ, higher scores indicate more positive overall attitudes after reverse-scoring the positive factor. Caregivers reported a mean psychological resilience score of 75.84 (SD = 13.68) and a mean caring attitudes score of 30.37 (SD = 5.55). The average score for abuse risk was 4.00 (SD = 2.82). The median score was 4, and the interquartile range (IQR) ranged from 1 to 6. Based on the pre-specified cutoff (CASE ≥ 3), 88 caregivers (66.2%) screened positive for higher abuse risk, while 45 (33.8%) scored below this threshold, indicating lower risk. Detailed descriptive statistics for these variables are provided in Table 2.

**Table 2:** Scores of psychological resilience, caring attitudes, and abuse risk among family caregivers of people with dementia (*N* = 133; mean ± SD, scores).

Variable	Score	Item average score
Psychological resilience	75.84 ± 13.68	3.03 ± 0.55
Strength	25.41 ± 4.58	3.18 ± 0.57
Tenacity	39.48 ± 8.11	3.04 ± 0.62
Optimism	10.95 ± 2.01	2.74 ± 0.50
Caring attitudes	30.37 ± 5.55	2.34 ± 0.43
Resistance factor	4.81 ± 1.22	2.41 ± 0.61
Burnout factor	9.33 ± 2.23	2.33 ± 0.56
External emotional factor	4.52 ± 1.04	2.26 ± 0.52
Negative factor	4.69 ± 1.30	2.35 ± 0.65
Positive factor	7.02 ± 1.49	2.34 ± 0.50
Abuse risk	4.00 ± 2.82	0.50 ± 0.35

SD, standard deviation.

### 3.3 Correlation between caring attitudes, psychological resilience, and abuse risk

Significant correlations were found among the main study variables. Psychological resilience was positively correlated with overall caring attitudes (*r* = 0.57, *p* < 0.001). Both psychological resilience (*r* = −0.43, *p* < 0.001) and caring attitudes (*r* = −0.71, *p* < 0.001) were negatively correlated with abuse risk. An examination of subscales revealed that the resistance dimension of caring attitudes showed the strongest positive correlation with resilience (*r* = 0.50, *p* < 0.001), while the negative (*r* = −0.63, *p* < 0.001) and burnout (*r* = −0.62, *p* < 0.001) dimensions of caring attitudes were most strongly associated with abuse risk. It should be noted that for the negative and burnout dimensions, higher scores indicate more positive attitudes (i.e., lower negativity and less burnout); therefore, the negative correlations with abuse risk indicate that more positive attitudes are associated with lower abuse risk. Within the psychological resilience scale, its three dimensions (optimism, strength, tenacity) were highly inter-correlated (all *r* > 0.65, *p* < 0.001). The five dimensions of caring attitudes also showed significant, though moderate, inter-correlations. These positive correlations are theoretically expected, as the subdomains within each scale reflect different facets of the same underlying construct. The cross-scale correlations (e.g., between resilience and caring attitudes) suggest conceptual overlap without complete redundancy, supporting the discriminant validity of the measures. Complete correlation coefficients are presented in Table 3.

**Table 3:** Correlation analysis of psychological resilience, caring attitudes, and abuse risk among family caregivers of people with dementia (*N* = 133, *r*).

Variable	Psychological resilience	Optimism	Strength	Tenacity	Caring attitudes	Resistance factor	Burnout factor	External emotional factor	Negative factor	Positive factor	Abuse risk
Psychological resilience	1										
Optimism	0.77***	1									
Strength	0.93***	0.71***	1								
Tenacity	0.97***	0.65***	0.83***	1							
Caring attitudes	0.57***	0.47***	0.54***	0.55***	1						
Resistance factor	0.50***	0.42***	0.46***	0.47***	0.71***	1					
Burnout factor	0.46***	0.32***	0.44***	0.44***	0.87***	0.48***	1				
External emotional factor	0.33***	0.29***	0.28***	0.32***	0.75***	0.42***	0.65***	1			
Negative factor	0.47***	0.41***	0.44***	0.45***	0.77***	0.56***	0.61***	0.38***	1		
Positive factor	0.41**	0.36***	0.39***	0.38***	0.65***	0.31***	0.38***	0.43***	0.35***	1	
Abuse risk	−0.43***	−0.42***	−0.42***	−0.38***	−0.71***	−0.47***	−0.62***	−0.54***	−0.63***	−0.41***	1

*** indicates *p* < 0.001.

### 3.4 Mediating role of psychological resilience between caring attitudes and abuse risk

To control for multiple comparisons across the five separate mediation analyses, a Bonferroni correction was applied, setting the significance threshold at *α* = 0.01 (0.05/5). To examine the mediating role of psychological resilience, we performed a mediation analysis using Model 4 of the PROCESS macro, with all variables standardized. Bias-corrected bootstrap confidence intervals were estimated with 5,000 resamples (Preacher and Hayes, 2004). The analysis revealed significant total effects of all caring attitude dimensions on abuse risk: positive factors (*β* = −0.36, *t* = −4.30, *p* < 0.001), resistance factors (*β* = −0.44, *t* = −5.40, *p* < 0.001), external emotional factors (*β* = −0.52, *t* = −6.45, *p* < 0.001), burnout factors (*β* = −0.57, *t* = −7.43, *p* < 0.001), and negative factors (*β* = −0.61, *t* = −8.58, *p* < 0.001).

The mediation analysis further indicated that psychological resilience significantly and negatively predicted abuse risk across all models: positive factors (*β* = −0.30, *t* = −3.27, *p* = 0.001), resistance factors (*β* = −0.25, *t* = −2.77, *p* = 0.006), external emotional factors (*β* = −0.26, *t* = −3.16, *p* = 0.002), burnout factors (*β* = −0.19, *t* = −2.32, *p* = 0.022), and negative factors (*β* = −0.17, *t* = −2.11, *p* = 0.037).

Each dimension of caring attitudes also had a significant positive effect on psychological resilience: positive factors (*β* = 0.36, *t* = 4.42, *p* < 0.001), resistance factors (*β* = 0.40, *t* = 5.01, *p* < 0.001), burnout factors (*β* = 0.40, *t* = 4.86, *p* < 0.001), negative factors (*β* = 0.38, *t* = 4.81, *p* < 0.001), and external emotional factors (*β* = 0.30, *t* = 3.54, *p* < 0.001). After accounting for the mediator, the direct negative effects of caring attitudes on abuse risk remained significant for all dimensions: positive factors (*β* = −0.26, *t* = −2.94, *p* = 0.004), resistance factors (*β* = −0.34, *t* = −3.91, *p* < 0.001), external emotional factors (*β* = −0.44, *t* = −5.40, *p* < 0.001), burnout factors (*β* = −0.50, *t* = −6.00, *p* < 0.001), and negative factors (*β* = −0.55, *t* = −7.14, *p* < 0.001).

**Figure 1:**
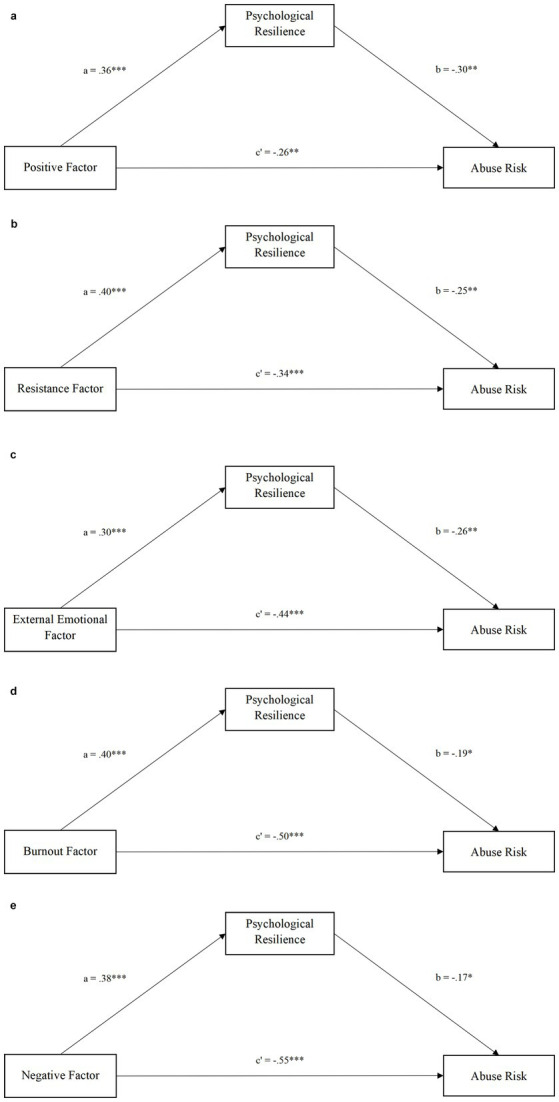
**(a)** Mediation model of positive factor on abuse risk via psychological resilience (*N* = 133). Path coefficients are standardized. *a*, *b*, and *c*′ represent *X* → *M*, *M* → *Y*, and *X* → *Y* paths, respectively. ***p < 0.001; **p < 0.01. Bootstrap with 5,000 resamples. **(b)** Mediation model of resistance factor on abuse risk via psychological resilience (*N* = 133). Path coefficients are standardized. *a*, *b*, and *c*′ represent *X* → *M*, *M* → *Y*, and *X* → *Y* paths, respectively. ***p < 0.001; **p < 0.01. Bootstrap with 5,000 resamples. **(c)** Mediation model of external emotional factor on abuse risk via psychological resilience (*N* = 133). Path coefficients are standardized. *a*, *b*, and *c*′ represent *X* → *M*, *M* → *Y*, and *X* → *Y* paths, respectively. ***p < 0.001; **p < 0.01. Bootstrap with 5,000 resamples. **(d)** Mediation model of burnout factor on abuse risk via psychological resilience (*N* = 133). Path coefficients are standardized. *a*, *b*, and *c*′ represent *X* → *M*, *M* → *Y*, and *X* → *Y* paths, respectively. ***p < 0.001; **p < 0.05. Bootstrap with 5,000 resamples. **(e)** Mediation model of negative factor on abuse risk via psychological resilience (*N* = 133). Path coefficients are standardized. *a*, *b*, and *c*′ represent *X* → *M*, *M* → *Y*, and *X* → *Y* paths, respectively. ***p < 0.001; **p < 0.05. Bootstrap with 5,000 resamples.

Furthermore, the bootstrap confidence intervals for both the direct and indirect (mediating) effects did not include zero (see Table 4), confirming that psychological resilience partially mediated the relationship between each caring attitude dimension and abuse risk. As a sensitivity analysis, we repeated the analysis using a binary outcome of abuse risk (CASE ≥3 vs. <3) with binomial logistic regression. More positive caring attitudes were significantly associated with lower odds of abuse risk (OR = 0.11, 95% CI [0.04, 0.27], *p* < 0.001). This result is consistent with the primary continuous-variable analysis and supports the robustness of the findings. Sensitivity analyses controlling for dementia stage as a covariate confirmed that the indirect effects of all five attitude dimensions remained significant. After Bonferroni correction, all a-paths, direct effects (*c*′), and indirect effects remained significant (all *p* < 0.001). The *b*-paths for the burnout factor (*p* = 0.022) and the negative factor (*p* = 0.037) did not reach the adjusted threshold; however, the overall pattern of mediation remained consistent across dimensions. The hypothesized mediation models were tested using PROCESS Model 4 (see Table 5 for complete regression statistics). The proportion of the partial mediating effect mediated by resilience varied across different attitude dimensions, ranging from 10.5% for negative factors to 29.2% for positive factors. Table 4 provides the complete decomposition of total, direct, and indirect effects for all five dimensions, along with their corresponding confidence intervals. The structural relationships of the mediation model are illustrated in Figures 1a–e.

**Table 4:** Decomposition of total effects, direct effects, and mediation effects (*N* = 133).

Pathway	Effect type	Effect value	Standard error	95% CI		
				Lower limit	Upper limit	Relative effect value (%)
Positive factor → psychological resilience → abuse risk	Total effect	−0.36***	0.08	−0.53	−0.20	—
	Direct effect	−0.26**	0.09	−0.43	−0.08	70.8
	Mediation effect	−0.11	0.04	−0.21	−0.04	29.2
Resistance factor → psychological resilience → abuse risk	Total effect	−0.44***	0.08	−0.61	−0.28	—
	Direct effect	−0.34***	0.09	−0.52	−0.17	77.4
	Mediation effect	−0.10	0.04	−0.19	−0.03	22.6
External emotional factor → psychological resilience → abuse risk	Total effect	−0.52***	0.08	−0.67	−0.36	—
	Direct effect	−0.44***	0.08	−0.60	−0.28	84.9
	Mediation effect	−0.08	0.03	−0.15	−0.03	15.1
Burnout factor → psychological resilience → abuse risk	Total effect	−0.57***	0.08	−0.72	−0.42	—
	Direct effect	−0.50***	0.08	−0.66	−0.33	86.6
	Mediation effect	−0.08	0.04	−0.16	−0.02	13.4
Negative factor → psychological resilience → abuse risk	Total effect	−0.61***	0.07	−0.75	−0.47	—
	Direct effect	−0.55***	0.08	−0.70	−0.40	89.5
	Mediation effect	−0.06	0.03	−0.13	−0.01	10.5

*p < 0.05, **p < 0.01, ***p < 0.001. Bootstrap confidence intervals for indirect effects are bias-corrected; significance is indicated by the CI excluding zero.

**Table 5:** Regression statistics for the mediation models (PROCESS model 4) (*N* = 133).

Predictor variable	Outcome variable	*R^2^*	*F*	Predictor in equation	*β*	SE	*t*	*p*	LLCL	ULCL
Positive factor										
Positive factor	Psychological resilience	0.30	5.84	Positive factor	0.36***	0.08	4.42	<0.001	0.20	0.51
Positive factor and psychological resilience	Abuse risk	0.30	5.12	Positive factor	−0.26**	0.09	−2.94	0.004	−0.43	−0.08
				Psychological resilience	−0.30**	0.09	−3.27	0.001	−0.48	−0.12
Resistance factor										
Resistance factor	Psychological resilience	0.33	6.61	Resistance factor	0.40***	0.08	5.01	<0.001	0.24	0.56
Resistance factor and psychological resilience	Abuse risk	0.33	6.00	Resistance factor	−0.34***	0.09	−3.91	<0.001	−0.52	−0.17
				Psychological resilience	−0.25**	0.09	−2.77	0.006	−0.43	−0.07
External emotional factor										
External emotional factor	Psychological resilience	0.26	4.89	External emotional factor	0.30***	0.08	3.54	<0.001	0.13	0.47
External emotional factor and psychological resilience	Abuse risk	0.39	7.85	External emotional factor	−0.44***	0.08	−5.40	<0.001	−0.60	−0.28
				Psychological resilience	−0.26**	0.08	−3.16	0.002	−0.42	−0.10
Burnout factor										
Burnout factor	Psychological resilience	0.32	6.41	Burnout factor	0.40***	0.08	4.86	<0.001	0.23	0.56
Burnout factor and psychological resilience	Abuse risk	0.42	8.75	Burnout factor	−0.50 ***	0.08	−6.00	<0.001	−0.66	−0.33
				Psychological resilience	−0.19*	0.08	−2.32	0.022	−0.36	−0.03
Negative factor										
Negative factor	Psychological resilience	0.32	6.35	Negative factor	0.38***	0.08	4.81	<0.001	0.23	0.54
Negative factor and psychological resilience	Abuse risk	0.47	10.73	Negative factor	−0.55***	0.08	−7.14	<0.001	−0.70	−0.40
				Psychological resilience	−0.17*	0.08	−2.11	0.037	−0.33	−0.01

*p < 0.05, **p < 0.01, ***p < 0.001, Bootstrap with 5,000 resamples. LLCI and ULCI refer to 95% bias-corrected bootstrap confidence intervals. All variables were standardized before analysis.

## 4 Discussion

The caregivers in our study demonstrated a moderate to high level of psychological resilience, which warrants consideration within the specific context of familial dementia care in China. This finding is consistent with some prior caregiver research (Duangjina et al., 2025; Jia et al., 2025), yet the scores observed were notably higher than certain domestic norms and reports from other caregiving contexts (Zhao T. et al., 2023). In this cross-sectional sample, this relatively robust resilience may reflect a psychological resource cultivated within and perhaps necessitated by the high-demand caregiving environment described in our introduction—one defined by filial obligation (xiao) and often lacking systemic support (Chen et al., 2025; Yu et al., 2025). It may also reflect a form of culturally-informed endurance (rennai) developed to manage chronic stress and uphold family duty (Yu et al., 2025; Xiang et al., 2025; Kim et al., 2016; Chaudhuri and Gupta, 2025). Sample characteristics may have contributed to this capacity, including longer caregiving durations that allow for the development of experience-based coping and higher educational attainment, which can facilitate problem-solving and access to information (Sin et al., 2025). Notably, nearly 40% of the present sample had been caregiving for less than 1 year, which suggests that resilience could partially reflect pre-existing traits or rapid adaptation rather than solely accumulated experience. In addition, caregivers of early-stage patients had significantly lower abuse risk than those of middle- and late-stage patients, a cross-sectional finding consistent with the hypothesis that disease progression is associated with increased caregiver burden (LeRolland et al., 2025; Yuan et al., 2024). This level of baseline resilience in our sample is crucial for interpreting the subsequent mediation analysis; it provides a substantive foundation for examining cross-sectionally how this resource is associated with caregivers’ attitudinal appraisals to influence behavioral risk, addressing the core gap identified in our introduction (Chen et al., 2025; Mei et al., 2025). Because the data are cross-sectional, these interpretations are hypothesis-generating and require replication in longitudinal studies.

In parallel, caregivers reported an overall moderate level of caring attitudes, a finding that highlights the complex emotional landscape within the familial caregiving role in China. This aligns with research indicating that the sustained, high-burden nature of dementia care is often associated with significant emotional strain, including burnout, which is related to more negative appraisals of the caregiving role (Lv et al., 2025; Im et al., 2025). Crucially, our dimensional analysis revealed that within this moderate global score, specific negative components—particularly feelings of burnout and resistance—were pronounced. This granular finding resonates with the tension inherent in the caregiving context outlined in our introduction: the cultural imperative of xiao coexists with a reality of solitary, high-stress care provision that lacks sufficient support (Chen et al., 2025; Mei et al., 2025). The prominence of these specific emotional states (e.g., exhaustion, resentment) suggests not a general negative disposition, but targeted areas of intense psychological friction where the ideal of devoted care conflicts with daily hardships. Understanding this attitudinal profile—characterized by moderate overall scores with peaks in specific distress domains—is essential for contextualizing cross-sectionally how these appraisals are associated with psychological resilience to influence behavioral outcomes, which is the central focus of our mediation model.

Most critically, the study revealed a high prevalence of abuse risk, with 66.2% of caregivers screening positive—a finding that underscores the pressing nature of this hidden crisis within home-based dementia care in China. The average risk score (4.00 ± 2.82) indicates a moderate overall level, but the high positive screen rate suggests widespread vulnerability. This proportion is notably higher than that reported in some prior domestic studies (Chen et al., 2019), a discrepancy that may reflect the intense, unsupported caregiving burden characteristic of the population we sampled—one navigating the pressures of filial duty with minimal formal support, as described in our introduction (Chen et al., 2025; Mei et al., 2025). This elevated risk cannot be viewed in isolation; it is cross-sectionally associated with the negative emotional states (reflected in lower scores on the burnout and resistance dimensions) identified in caregivers’ attitudes. The convergence of a culturally mandated yet under-supported care model, measurable emotional strain in key attitudinal domains, and a high rate of behavioral risk signals a critical need to examine the statistical indirect associations—such as the potential role of resilience—that link caregivers’ internal experiences to their behavioral outcomes. This finding directly justifies the core analytical focus of our study, which is to describe these statistical associations.

The analysis revealed a significant positive correlation between caregivers’ psychological resilience and their caring attitudes, cross-sectionally indicating that greater resilience is associated with more positive and adaptive appraisals of the caregiving role. This finding aligns with prior research by Zheng et al. (2023), supporting the notion that resilience may foster a more constructive outlook toward caregiving challenges over time, though cross-sectional data cannot establish directionality. Critically, this correlation provides empirical grounding for the Stress Process Model (Han et al., 2022), which was applied in our study, suggesting that internal resources (resilience) are intrinsically linked to primary cognitive-emotional appraisals (attitudes) in this sample. This relationship establishes the necessary precondition for examining our proposed mediation model cross-sectionally. We applied a Bonferroni correction (*α* = 0.01) to control for multiple comparisons. All a-paths, direct effects (*c*′), and indirect effects remained significant (*p* < 0.001). However, the b-paths for the burnout factor (*p* = 0.014) and the negative factor (*p* = 0.030) did not reach the adjusted significance threshold. This suggests that for these two dimensions, the mediating role of psychological resilience may operate primarily through the indirect pathway rather than through the direct effect of resilience on abuse risk. Nevertheless, the overall pattern of mediation remained consistent across dimensions, supporting a partial mediating role of resilience.

Furthermore, both caring attitudes and psychological resilience demonstrated significant negative correlations with abuse risk. Cross-sectionally, this suggests that these psychological resources are associated with better stress management and a lower likelihood of negative behavioral outcomes. The result is consistent with work by Xiao et al. (2014) within similar cultural contexts. A key granular finding was that the burnout and negative factors within the attitude construct showed the strongest inverse relationships with abuse risk. Given that higher scores on these dimensions indicate more positive attitudes (i.e., lower burnout and negativity), these inverse relationships indicate that caregivers with lower scores (i.e., higher burnout and negativity) exhibit greater risk in cross-sectional analyses. This cross-sectional finding suggests that specific, intense negative emotional states—rather than a generally negative disposition—are associated with higher abuse risk. If confirmed in longitudinal studies, interventions that help caregivers manage these acute forms of distress could be important for risk reduction.

The core contribution of this study lies in demonstrating a nuanced, differential indirect association. To examine this association, we analyzed the indirect effects separately for each dimension of caring attitude, given their relatively low inter-correlations compared to the strong internal coherence of the psychological resilience dimensions. This approach indicated that psychological resilience shows a significant partial indirect association in the overall relationship, consistent with the theoretical proposition that internal resources may translate appraisals into outcomes (Sun et al., 2024) and with global evidence on resilience’s protective associations (Rocks et al., 2025). After adjusting for demographic covariates (age, gender, education, income, caregiving duration, relationship, assistance, and self-rated health), the indirect effects for all five dimensions remained significant, supporting the robustness of these associations.

The crucial novel finding is a substantial variation in indirect effect strength across attitudinal dimensions. For burnout and negative factors, the direct effect accounted for the majority of the total effect (86.6 and 89.5%, respectively), with the indirect effect via resilience being much smaller (13.4 and 10.5%). Although the b-paths for these two dimensions did not survive Bonferroni correction (*p* = 0.014 and *p* = 0.030), their indirect effects (*a* × *b*) remained significant (*p* < 0.001), indicating that the mediation operates mainly through the strong *a*-path (attitude → resilience). Here, lower scores on these dimensions (indicating higher burnout and more negative attitudes) were associated with higher abuse risk. This cross-sectional pattern suggests that acute emotional overwhelm is directly associated with higher abuse risk, with a smaller portion of the association operating through resilience. One interpretation is that when caregivers experience high levels of burnout or negativity, the emotional overwhelm may override any potential buffering effect of resilience—a hypothesis that requires longitudinal testing. Conversely, for positive and resistance factors, resilience showed a substantially stronger indirect association (29.2 and 22.6% of the total effect), highlighting its potential role in helping caregivers maintain meaning and manage reluctance. Thus, resilience may be more effective in attenuating abuse risk when caregivers already hold positive attitudes or struggle with reluctance, rather than when they are already emotionally overwhelmed.

This differential pattern extends our team’s prior person-centered work on high-stress caregiver profiles (Chen et al., 2025; Mei et al., 2025), suggesting a potentially modifiable psychological factor that varies with the caregiver’s emotional state. Notably, caregivers with higher resilience showed significantly lower abuse risk even when reporting less positive attitudes, a cross-sectional finding that points to resilience’s independent statistical buffering association. Within China’s high-demand and under-supported familial dementia care context, these findings suggest that resilience is directly associated with lower abuse risk and also shows an indirect association through caregiving appraisals, offering a hypothesis-generating target for abuse prevention interventions (Yu et al., 2025; Wang et al., 2025; Cheung et al., 2020). If these associations are causal, interventions that strengthen resilience could be particularly beneficial for caregivers who struggle with maintaining positive attitudes. Unlike Pan et al. (2023), who focused on caregiver burden and coping styles, our study uses multi-dimensional caring attitudes and reveals dimension-specific indirect effects (ranging from 10.5 to 29.2%), providing a more nuanced target for intervention.

Our findings offer some hypothesis-generating ideas for theory, practice, and policy by describing statistical indirect associations rather than proven mechanisms. Theoretically, they highlight the complexity of the attitude-behavior link and support the potential value of dual-focus interventions that simultaneously address acute emotional distress and build foundational resilience (Kwok et al., 2025; Santonja-Ayuso et al., 2024). In practice, our cross-sectional results hint that a dimension-sensitive approach might be helpful (Chi et al., 2024). For caregivers experiencing acute burnout or negativity—states cross-sectionally linked to the dominant direct association in our model—immediate support (e.g., respite care, crisis counseling) might help de-escalate risk. But this is only a hypothesis; we do not know if it actually works. Concurrently, strength-based resilience-building programs (e.g., cognitive reframing, emotional regulation) could enhance the psychological resource, which showed a stronger indirect association with lower abuse risk for attitudinal challenges such as resistance or loss of purpose. However, because our data are cross-sectional, these suggestions are speculative. At the policy level, support could move beyond rhetoric to fund culturally adapted programs and address structural stressors—such as low income and a lack of assistance (Chen et al., 2025; Li et al., 2025)—that cross-sectionally correlate with lower resilience and more negative attitudes. A multi-level approach that combines individual psychological support with structural enablers (e.g., role-sharing mechanisms, and micro-subsidies) might achieve synergistic risk reduction, but this needs to be tested in real-world experiments. None of these recommendations should be taken as proven; they are directions for future research.

## 5 Limitations and future research directions

Several limitations of this study must be acknowledged. First, its cross-sectional design precludes definitive causal inferences regarding the identified mediation pathways, despite their grounding in established theory. Second, although the sample size was adequate for the analyses conducted, and the response rate was 48.01%, the practical challenges of recruiting family dementia caregivers resulted in a sample that is not large; future research would benefit from larger, multi-center cohorts to enhance the robustness and generalizability of the findings. Although post-hoc power analysis indicated adequate power (0.82–0.89), some methodological guidelines recommend a larger sample size for complex mediation models with bootstrapping. Therefore, our findings should be considered preliminary and require replication in larger cohorts. Third, the use of a convenience sample from a single region may limit the broader applicability of the results to all caregiver populations in China. Fourth, the dementia stage was based on conventional clinical categories rather than continuous measures, which may have reduced information sensitivity. Fifth, we did not collect data on several other potential confounders, including functional dependence, neuropsychiatric symptoms of the care recipient, caregiver burden, caregiver depression/anxiety, and social support. Their possible influence on the observed associations cannot be ruled out. Sixth, reliance on self-report measures may introduce common method bias and social desirability bias, especially for sensitive questions about abuse risk. Although we used anonymity, neutral wording, reverse-scored items, and interviewer-administered questionnaires for participants with limited literacy, these biases cannot be completely ruled out. Comprehension issues also remain a possibility. Future longitudinal, multi-center studies with larger samples and more comprehensive confounder assessment are needed to validate our exploratory findings.

## 6 Conclusion

Addressing an important need for dementia care research in Asian cultural contexts, this study focuses on a meaningful research question: whether psychological resilience is associated with caring attitudes and abuse risk among Chinese family dementia caregivers. The findings suggest that resilience shows an indirect association with abuse risk via caring attitudes, and this indirect effect varies substantially across emotional dimensions of caring attitudes. Specifically, the association from acute emotional states (e.g., burnout, high negativity) to abuse risk is predominantly direct, whereas resilience shows a stronger indirect association for dimensions related to maintaining a positive outlook and managing reluctance. These results provide valuable take-away messages about how cultural practices (e.g., filial piety) and support gaps may relate to caregiver risk. However, given the cross-sectional design, these findings should be interpreted as exploratory and hypothesis-generating. Longitudinal and intervention studies are needed to establish causal direction and clinical utility. Within China’s high-demand, under-supported familial dementia care context, this evidence provides a preliminary, culturally grounded basis for developing support strategies, pending further validation.

## Data Availability

The raw data supporting the conclusions of this article will be made available by the authors, without undue reservation.

## Footnotes

1
https://www.wjx.cn/
